# The development of an occupational therapy intervention for adults with a diagnosed psychotic disorder following discharge from hospital

**DOI:** 10.1186/s40814-018-0267-7

**Published:** 2018-04-23

**Authors:** Mary Birken, Claire Henderson, Mike Slade

**Affiliations:** 1 School of Health Sciences, City, University of London, Northampton Square, London, EC1V 0HB UK; 2 Health Service & Population Research Department, Institute of Psychiatry, Psychology & Neuroscience, King’s College London, London, UK; 3 School of Health Sciences, University of Nottingham, Nottingham, UK

**Keywords:** Community mental health, Hospital transition, Leisure activities, Productive occupations, Self-care

## Abstract

**Background:**

A deterioration in mental health and admission to an acute mental health unit can result in skill loss and decreased participation in daily life. Furthermore, discharge from hospital is associated with high risks of social isolation and suicide. This intervention development study aims to describe the rationale, methods and processes of developing an intervention for adults with a diagnosed psychotic disorder following discharge from hospital. The intervention aims to increase participation in self-care and leisure, wellbeing and quality of life and reduce crisis service use.

**Methods:**

The UK Medical Research Council framework for the development of complex interventions was used to guide the process of developing the intervention to ensure the developed intervention is empirically justifiable and evidence based. The development involved a systematic and literature reviews and focus groups with people with psychosis and clinical staff to understand the problems the intervention should address and approaches to resolving these.

**Results:**

A manualised 4-month intervention named Graduating Living skills Outside the Ward (GLOW) was developed for use by occupational therapists for people with a diagnosed psychotic disorder following discharge from hospital. The one-to-one stepped intensity intervention is of 4 months in duration and takes place in the person’s home and in community locations. The intervention aims to increase occupational performance of domestic and personal self-care, leisure and some productive roles.

**Conclusions:**

The intervention developed in this study has potential to improve the efficiency of community mental health services following discharge from hospital as it is evidence-based, time-limited and manualised and aims to reduce hospital admissions and crisis service use. The intervention will be tested to assess its clinical and cost effectiveness in a randomised controlled trial.

**Electronic supplementary material:**

The online version of this article (10.1186/s40814-018-0267-7) contains supplementary material, which is available to authorized users.

## Background

The current estimated prevalence of psychotic disorders in the UK is 0.7% [1], and approximately 220,000 people are treated for schizophrenia in the UK by the NHS at any one time [2]. Having schizophrenia is associated with high levels of unemployment [3], discrimination and social isolation [4]. It is the most common diagnostic group to be admitted to acute mental health inpatient services [5]. An estimated 42,000 people with a diagnosed psychotic disorder are admitted to hospital each year with a median length of stay of 38 days [6]. Therefore, having a psychotic illness is costly due to the associated high use of services and high rates of unemployment.

On discharge from an acute mental health hospital, people can experience social isolation, financial problems and difficulty looking after themselves [7]. Also, the period following discharge from hospital is a period of high risk of suicide [8]. Furthermore, losing a job, having relationship problems and living alone have been found to be risk factors for committing suicide following discharge from hospital [9]. This supports the view that being able to take part in daily life is important for health and recovery from a relapse following discharge from hospital.

Unmet needs of people with mental health problems post-discharge from an acute mental health ward have been identified regarding day time activities (26%, *n* = 45), food (12%, *n* = 20) and looking after their home (5%, *n* = 5) [10]. Additionally, people with a diagnosed mental health problem reported that they experienced stigma, had low self-esteem, avoided social contact and lost friendships following discharge from hospital [11]. Furthermore, service users have identified that there is a lack of follow-up after discharge regarding daily activities, and that a stepped approach to discharge would support their return to cooking and domestic activities [12].

The need to use a more proactive approach to reduce the long-term impact of mental ill-health for the individual and to reduce costs for the NHS has also been emphasised by the government in their 5-year forward view for mental health document published in 2016 [3]. Currently, intervention post-discharge from an acute mental health ward in the UK routinely focus on medication and monitoring of mental health symptoms, and includes crisis and home treatment team and/or a 7-day follow-up visit or phone call [13, 14]. An intervention, following discharge from hospital to support participation in daily activities, has the potential to improve the transition by supporting the reorganisation of daily tasks in a timely manner, reducing relapse in mental illness and readmissions to hospital. Receiving such an intervention also has the potential to reduce long-term disability and increase quality of life and wellbeing. This paper reports the process of developing an intervention called GLOW (Graduating Living skills Outside the Ward), for people with a diagnosed psychotic disorder post-discharge from hospital to increase self-care and leisure and increase wellbeing and quality of life. This study lays the foundation for feasibility and piloting of the developed intervention by providing a transparent and comprehensive report of the method and process of the development of an evidence-based intervention.

Occupational therapists hold skills and knowledge regarding increasing participation in daily activities and are therefore best placed to provide such interventions post-discharge from hospital as part of multidisciplinary community mental health care. Currently, occupational therapists may work with people with a diagnosed mental illness post-discharge from hospital, using their unique tacit knowledge, published theories and models of practice [15]. However, there is no evidence regarding the effectiveness of occupational therapy in this context [16] or published best practice. An evidence-based intervention manual would support assessment of the clinical and cost effectiveness of the occupational therapy intervention for people with a diagnosed mental illness, an identified priority by clinicians, managers and health service commissioners [17–19].

People with a diagnosed psychotic disorder have severe problems in more areas of occupational performance than adults with non-psychotic disorders [20] and high rates of admission; therefore, the intervention targets people with a diagnosed psychotic disorder.

The aim of this intervention development study was to describe the rationale, methods and processes used to develop an intervention for adults with a diagnosed psychotic disorder following discharge from hospital. The process of developing the intervention will be described in the next section, and the developed intervention will be outlined in the “Results” section.

## Methods

The development phase of the UK Medical Research Council framework for developing and evaluating complex interventions was used to guide the intervention development process [21].

**Fig. 1:**
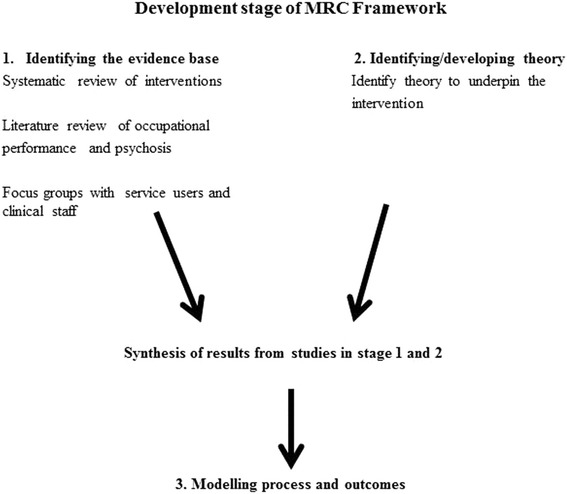
Use of the three stages of the development phase in this study

This phase consists of three stages: (1) identifying the evidence base of the intervention, (2) identifying/developing theory to underpin the intervention and (3) modelling the process of the intervention and outcomes. Figure 1 outlines the use of the three stages in this study.

### Stage one: Identifying the evidence base

Figure 1 describes the three studies that were conducted in stage one to contribute to the evidence base of the intervention. The studies involved in identifying the evidence base of the intervention will now be described in more detail.

#### Systematic review of interventions to improve occupational performance of everyday activities following discharge from hospital

The rationale for the review, review questions and methods are outlined in Table 1.

**Table 1:** Summary of methods for systematic review

Review questions	1. To identify and characterise studies of interventions to improve occupational performance for adults with psychosis 2. To characterise the theory base, level of standardisation and treatment fidelity for identified interventions
Population	Participants aged between 18 and 65 with a diagnosis of schizophrenia or schizoaffective disorder (or at least 70% of sample) living in the community.
Intervention	Studies of interventions which primarily aim to improve occupational performance, excluding vocational occupations.
Outcomes	The primary outcome is occupational performance
Types of studies	Randomised controlled trials, quasi-experimental studies, observational studies, and qualitative studies.
Data sources	Three data sources were used: online databases, internet search and a hand search. Online database searches were carried out in January 2011. The online databases searched were CINAHL, PsycINFO, MEDLINE, ASSIA and Embase databases, the Cochrane Library, OTCATS (Occupational Therapy Critically Appraised Topics), OTseeker, and online databases of unpublished PhD and Masters’ theses available from the College of Occupational Therapy and the Institute of Psychiatry. The internet search using Google was carried out using the same search terms that were used in databases, to identify grey literature and conference proceedings relating to psychosis and occupational performance. The table of contents of the following publications were also hand searched: Schizophrenia Bulletin, Schizophrenia Research, Psychiatric Services, British Journal of Occupational Therapy, Occupational Therapy in Mental Health and Occupational Therapy Journal of Rehabilitation.
Search terms	Population: Schizophrenia OR Psychotic disorders OR Affective disorders, Psychotic OR Severe and enduring mental health AND community Intervention: Leisure activities OR functional status OR community integration OR self-care OR activities of daily living OR skill acquisition OR skill retention OR meal preparation OR occupational performance OR occupation* AND performance OR participation OR engagement OR functioning OR impairment or adaptation Design: (Intervention AND (study OR evaluation)) OR Pilot study OR Randomised Controlled Trial Outcome: Change in occupational performance OR skill acquisition
Search strategy	A search using variations of the above terms appropriate to the terms used in the databases was carried out in online databases and mapped and unmapped against indexed subject heading where this option exists. The search strategy was restricted to studies published since 1995 until January 2011 and English language only.
Study selection	Abstracts of identified papers were assessed for eligibility. For studies not excluded on abstract, the full paper was obtained and assessed in more detail against the inclusion and exclusion criteria. A second rater was used to independently assess the eligibility of a random selection of 20% of full papers, to estimate concordance on inclusion.
Quality assessment	Appraisal of quantitative methodologies was carried out using the Quality Assessment tool for quantitative studies [43]. A cut-off score was not used as an inclusion criterion.
Data analysis	Narrative synthesis was used to integrate the results of studies included in the review, as the studies were clinically diverse, used a range of outcome measurement tools and were shown to have a range of comparisons. The narrative synthesis used in this study consisted of three stages as described by Roberts [44].

**Fig. 2:**
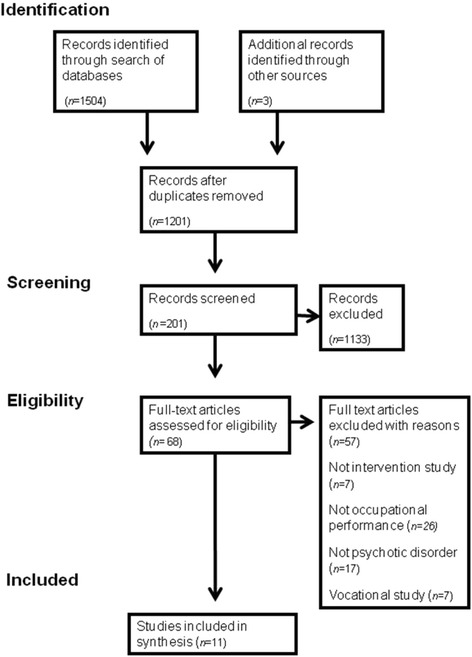
Flow diagram of studies in the systematic review

Figure 2 presents the PRISMA flow diagram for the study.

A summary of the 11 studies included can be found in Additional file 1. No studies were found of interventions that focused specifically on improving occupational performance following discharge from hospital. Therefore, a new intervention needed to be developed that focused on that context.

#### Literature review of occupational performance for people with psychosis

The first aim of the literature review was to identify studies exploring characteristics of occupational performance of people with a diagnosed psychotic disorder. The second aim was to identify what is known regarding the problems and facilitators that people with a diagnosed psychotic disorder experience regarding occupational performance of leisure and self-care. This enables the intervention to target the identified problematic aspects of occupational performance and have the greatest impact on improvement [22]. A summary of methods used in the literature review and results are in Table 2.

**Table 2:** Summary of literature review methods

Search terms	((Occupation* AND (functioning OR impairment OR adaptation OR engagement OR performance)) OR participation in daily life OR (Function* AND (performance OR recovery OR status OR outcomes OR skills) OR activities of daily living OR independent living skills OR psychosocial functioning OR community living skills OR self-care OR leisure) AND (psychosis OR psychotic illness OR schizophrenia OR schizoaffective OR severe and enduring mental health)
Online databases searched	CINAHL, Embase, PsycINFO and MEDLINE
Inclusion criteria	A focus on occupational performance of adults with a diagnosed psychotic disorder or where at least 70% of the participants had a diagnosed psychotic disorder and between the ages of 18–65. Qualitative and quantitative studies were included.
Quality assessment	The RATS (Relevance, Appropriateness, Transparency, Soundness) qualitative research review guideline was used to evaluate qualitative studies [45]. The quality assessment tool for quantitative studies developed by Effective Public Health Practice Project was used to rate the quality of included quantitative studies [43]. All studies were included in the review following quality assessment.
Results	Twenty-three papers meeting the inclusion criteria were found in the review. Ten of the studies used qualitative methods and 13 used quantitative methods to investigate aspects of occupational performance for adults with psychosis. No studies focused on post-discharge from hospital. Key findings The literature review identified that people with psychosis with higher levels of information-processing skills were more independent in their living skills. Reduced motor and process skills impacted on their ability to carry out daily activities, in addition to skill development being disrupted at onset of psychosis. People with psychosis have been identified as being under occupied and describe a lack of routine and problems with personal activities of daily living. A lack of social contacts has been identified as a barrier to leisure.

The literature review found both qualitative and quantitative studies relating to occupational performance of people with a diagnosed psychotic disorder but found no studies focusing on occupational performance following discharge from hospital; therefore, new knowledge was required and was generated in the focus groups. The results of the literature review did contribute to the developed manualised intervention to compliment the information regarding occupational performance specific to post-discharge from hospital.

#### Focus groups with service users with psychosis

Service users’ hold knowledge of themselves in relation to their occupations and their environment, and this knowledge is an essential source of evidence to inform an intervention [23] Focus groups were used to identify the problems of occupational performance post-discharge from hospital for people with psychosis from their perspective. Focus groups were used to make use of group dynamics to stimulate in-depth discussion of the topic [24]. It has been identified that using focus groups has advantages over individual interviews, particularly when there may be power differences between the participants and professionals [25]. In this instance, the participants have peer support and are more likely to be open in their replies to questions. Two focus groups with service users with psychosis who had been discharged from hospital in the previous 6 months took place with six service users participating in the focus groups. A summary of the methods used in this focus group are in Table 3.

**Table 3:** Summary of methods for the focus group with service users

Aim	To explore the experiences of people with a diagnosed psychotic disorder following discharge from hospital regarding their occupational performance in the areas of self-care and leisure.
Participants	Inclusion criteria: adults between ages of 18–65 with a primary diagnosis of a psychotic disorder, who have been discharged from hospital within the previous 3–6 months Exclusion criteria: being unable to give consent, too unwell to participate as identified by named clinician or requiring an interpreter to participate in the focus group
Focus group facilitator	A researcher with lived experience of using mental health services and being admitted to hospital asked the questions and a co-facilitator was present.
Topic guide	Can you all tell us what daily activities you spent your time doing, during the first month after you came out of hospital? And also any daily activities you wanted to do during this time but were unable? Can you all tell us what daily activities you spent your time doing, during the second and third month after you came out of hospital? And also any daily activities you wanted to do during this time but were unable? What activities were important for you to be able to do better, or more independently, during these first 3 months?
Recruitment	Clinicians from adult community mental health teams in one NHS trust were asked to identify and ask suitable participants if they would like to hear more about the project from the researcher and read the Participant Information Sheet before deciding to take part. The researcher met with those interested in hearing more about the study and the potential participants’ right to withdraw from the study, or to decline to take part without this affecting the care they receive was emphasised. The focus groups took place in a community mental health team to enable the participants to access support should they become distressed during the focus groups.
Analysis	Thematic analysis as outlined by Braun and Clark was used and analysis was driven by the research questions [46]. The trustworthiness [47] of the results was ensured through maintaining credibility through the inclusion of direct quotes in the final report. Member checking was not carried out as their perspective on the topic may have changed from those recorded closer to the time of discharge from hospital. Dependability of the findings was ensured by using a recognised form of data analysis.

The results of the focus groups identified the following themes: hesitancy to be more independent on discharge, the challenges of organising meals independently and difficulty in initiating identified community leisure activities. The final theme was the challenges of how to pace the building up level of activities over time.

#### Focus groups with clinical staff

Tacit and experiential knowledge held by clinical staff are important sources to explore when researching clinical practice and provide a source of evidence for intervention development [26]. Therefore, a focus group was held with occupational therapists and recovery support workers to seek their expert opinion that they developed in practice working with service users following discharge from hospital.

A summary of the methods used in this focus group are in Table 4.

**Table 4:** Summary of methods for the focus group with clinical staff

Aim	To gain the perspectives of clinical staff regarding their understanding the problems of occupational performance post-discharge from hospital and approaches they have used to address them.
Participants	Inclusion criteria: Occupational Therapists and Support, time and Recovery (STR) workers delivering interventions to address difficulties of occupational performance. Working in the community and recent experience (within last year) of working with individuals with a diagnosed psychotic disorder following discharge from hospital.
Facilitator	A researcher with lived experience of using mental health services and being admitted to hospital asked the questions and the author was co-facilitator.
Topic guide	*From discharge date to first month post discharge* From your experience of working with people with a diagnosed psychotic disorder following discharge from hospital what are the difficulties that you have observed they have had regarding their ability to carry out daily activities? What daily activities have service users told you are important to them during this first month? *From 2 months to 3 months post discharge* From your experience of working with people with a diagnosed psychotic disorder following discharge from hospital what are the difficulties that you have observed they have had regarding their ability to carry out daily activities? What daily activities have service users told you are important to them during this time? Can you describe some the ways in which you have supported people to improve their ability to carry out daily activities following discharge from hospital?
Recruitment	The clinical staff were recruited by inviting all Occupational Therapists and STR workers in one NHS trust who worked in community mental health teams for people with a diagnosed psychotic disorder, to participate in focus groups. The staff were given a copy of the Participant Information Sheet and given the opportunity to ask the researcher any questions, before deciding to take part in the study. They were given Consent Forms, which were signed by participants on the day of the focus group, before it commenced.
Analysis	Thematic analysis as outlined by Braun and Clark was used and analysis was driven by the research questions [46]. The trustworthiness [47] of the results was ensured through maintaining credibility through the inclusion of direct quotes in the final report. Member checking was not carried out as their perspective on the topic may have changed from those recorded closer to the time of discharge from hospital. Dependability of the findings was ensured by using a recognised form of data analysis.

The following themes were identified from the staff focus group which contained four participants: importance of therapeutic use of self, grading the activities, balancing a focus between basic self-care and long-term goals and focusing on the social and physical environments.

#### Synthesising the findings to inform the intervention

The findings of the focus groups were categorised into areas that an intervention should focus on, as prioritised by the service users and clinical staff, and approaches to addressing these, as also identified by service users and staff; this is outlined in Table 5.

**Table 5:** Summary of findings of stage one and two, and implications for intervention

Sub-study	Finding	Implication for intervention
Stage one		
Systematic review	No intervention found to improve occupational performance following discharge from hospital	Development of a new intervention
Literature review of psychosis and occupational performance	Problems with cognitive functioning, performance skills and knowledge, process skills	Intervention to address these areas by including the following: Assessment of volition, habituation and performance. Activity grading Developing self-knowledge of Occupational performance to enable ongoing work towards long-term goals post intervention.
	Social and physical environmental barriers	Assessment of home environment Environmental enhancements and considerations
	Meaningful Occupations	Identification of meaning of occupations through assessment and service user led goal setting
	Routine	Assessment of routine included in the intervention Activity grading
	No problems specific to post discharge have been identified.	Study needed to identify new information regarding occupational performance following discharge from hospital to inform the intervention.
Focus groups with service users and clinicians	Slowly picking up daily life again	Intervention to be provided over 4 months Intervention to include Activity grading
	Importance of therapeutic use of self	Use a model of therapeutic use of self in the intervention
	Balancing a focus between basic self-care and long-term goals	Stepped level of intensity to support basic self-care post discharge followed by longer term goals regarding leisure Developing self-knowledge of occupational performance to continue working towards long-term goals post intervention.
	Building a picture of the future whilst on the ward	Start intervention prior to discharge from hospital
	Reluctance to be more independent	Use of MOHO to increase motivation for occupation, and occupational performance Assessment of occupational performance Development of self-knowledge and self-management of own occupational performance
	Barriers to accessing leisure activities	Assessment of occupational performance included in the intervention Intervention to take place in relevant community venue Use of Interest Checklist
	Managing meals	Use of strategies to overcome physical barriers in home environment Use of activity grading as a component
Stage two		
Identifying/developing theory	Model of human occupation identified	Assessment tools based on MOHO to be used as required within intervention.

### Stage two: Identifying/developing theory

To explain the process by which the clinical intervention brings about change in the individual [21], a literature review of theory relating to occupational performance was completed. Criteria for selection of an appropriate theory were developed. The four criteria for the theory selection were as follows:

1. Describing the process of change in occupational performance
2. Demonstrates construct validity to ensure the theory is empirically defensible
3. Clinical assessment tools have been developed based on the model to enable the clinicians to apply the theory in a standardised way
4. Suitable for application with people with mental health problems

To identify relevant theory, a search of the literature was carried out using four sources:

1. An online search of CINAHL and MEDLINE databases. The search terms used were theory, theoretical knowledge, theory-practice relationship, theoretical AND occupational therap* OR activities of daily living OR leisure.
2. Online book catalogues of King’s College London and College of Occupational Therapists were searched.
3. A hand search of mental health and occupational therapy text books at the College of Occupational Therapists library.
4. Reference lists of the papers found in the above literature review and systematic review were hand-searched to identify additional published papers relating to theoretical knowledge of occupational performance.

Eight theoretical models were identified in the review. Only one met all the above criteria and was therefore suitable for use in underpinning the intervention, Model of Human Occupation (MOHO) [27] and will be described in the “Results” section.

Table 5 summarises the findings of the sub-studies in stage one and two and the implication for the content, structure and format of the intervention.

### Stage three: Modelling the process and outcome of the intervention

The findings of all the sub-studies were summarised to determine areas of occupations the intervention should address, what approaches identified should be used, and structure and format of the intervention, ahead of modelling the process and outcome of the intervention.

A causal modelling approach [21] was used to outline how the intervention components work together to bring about the planned outcome of the intervention, according to the chosen theoretical base of the intervention.

#### Manualising the intervention

Following completion of the development of the content of the intervention, it was then structured to form an intervention manual. The benefits of developing a manualised intervention include providing structure for an intervention’s delivery, enabling the intervention to be consistently carried out by different therapists, facilitating clinician training of the intervention and allowing intervention replication in different contexts [27]. In this study, the intervention manual was developed following the model outlined by Carroll and Nuro [28]. This model was used as it proposes a stage development of a manual in parallel to the research stages, that is, pilot/feasibility, and effectiveness trials, used to test a new intervention. The purpose of stage one manual development is to provide an initial specification of the treatment techniques, goals, and theoretical active ingredients and for use in a pilot or feasibility study [28].

In addition to the development of an intervention manual, fidelity to the intervention was enhanced, as recommended by Bellg and colleagues [27] by providing standardised training on use of the intervention, and ensuring skill acquisition during the training through active discussion of the occupational therapists’ role during GLOW. An intervention Fidelity checklist was developed to monitor the extent to which the clinicians adhered to the content of the intervention manual.

Following development of the manual it was sent to six clinicians, some of whom participated in the focus group and the facilitator of the focus groups, for comments on all aspects of the intervention, amendments were made to manual accordingly and resent to the group for further comments before finalising the content of the manual.

## Results

The Template for Intervention Description and Replication (TIDieR) checklist [29] was used to organise the description of the developed intervention. The developed intervention was named Graduating Living skills Outside the Ward (GLOW), by the service users who participated in the development of the intervention. GLOW is underpinned by a comprehensive understanding of occupational performance that is achieved during occupational therapy training; therefore, GLOW is designed to be delivered by occupational therapists. GLOW is a ten-session one-to-one intervention. It aims to increase occupational performance of self-care, leisure and productive roles that is, an improvement in the “doing” of these occupations following discharge from hospital, and in doing so increase wellbeing and quality of life. It is anticipated that these changes will reduce further relapses of mental ill-health and reduce admissions to hospital. GLOW does not target gaining paid employment, as existing evidence-based services using Individual Placement and Support (IPS) have been established already to support obtainment of paid employment [30].

The theoretical basis for the intervention is the Model of Human Occupation (MOHO) [31]. MOHO describes the process of changing occupational performance, studies have demonstrated it has construct validity [32–35], clinical assessment tools have been developed based on the model, and it is used in clinical practice in mental health settings. MOHO outlines the relationship between a person’s motives for occupation, habits and roles, and physical and cognitive performance capacities in the context of their environment [31].

A second model, the Intentional Relationship Model (IRM) [36], is included in the intervention; it will guide the therapeutic relationship developed by the clinician during the intervention.

The intervention is of stepped intensity with the first four sessions taking place on a weekly basis in the person’s home. The following five fortnightly sessions take place in community venues where planned activities will take place during the sessions; the final session takes place in the person’s home.

The sessions are designed to last approximately 1 h during the first four sessions and up to 45 min for the remainder of the sessions. The intensity of the duration is informed by the Intentional Relationship Model [36], in that the occupational therapists are required to reduce the degree to which they plan and lead the sessions over time. The content of the intervention sessions are based on the individual service users’ goals regarding participation in leisure and self-care.

GLOW has four components: (1) assessment of volition, habituation and performance capacity; (2) activity grading; (3) environmental enhancements and considerations; and (4) developing self-knowledge of occupational performance.

### Description of the four components of GLOW

#### Assessment of volition (motivation for occupation), habituation and performance capacity

The clinical assessment aims to understand the current and past performance capacities, motivation for occupation, habits and routines and future occupational goals of the individual. The assessment tools used are MOHOST (Model of Human Occupation Screening Tool) [35], REIS-SF (Residential Environment Impact Scale-Short Form) [37] and UK Modified Interest Checklist [38].

#### Activity grading

Activity grading consists of balancing the ability of the person with the task, to ensure the person can gradually complete the task independently over time.

#### Environmental enhancements and considerations

The environment can be defined as the particular physical and social, cultural, economic and political features of one’s context that impact upon the motivation, organisation and performance of occupation [31]. In GLOW, the occupational therapist assesses the home, local and community venue environments to identify the barriers and facilitators of occupational performance for the person.

#### Developing self-knowledge of occupational performance

This component consists of the occupational therapists, who hold knowledge regarding occupation and occupational performance, imparting this knowledge to service users to enable them to use this in their own lives beyond the duration of the intervention.

### Modelling the process and outcome of the intervention

Figure 2 outlines the mechanism of change expected within the intervention, using MOHO as the theoretical basis of the intervention, to bring about the expected outcome of the intervention, and in doing so, it is anticipated that the intervention will lead to improvements in wellbeing and quality of life, and reduce their need for hospital admissions and use of crisis services.

### Measuring the outcomes of GLOW

**Fig. 3:**
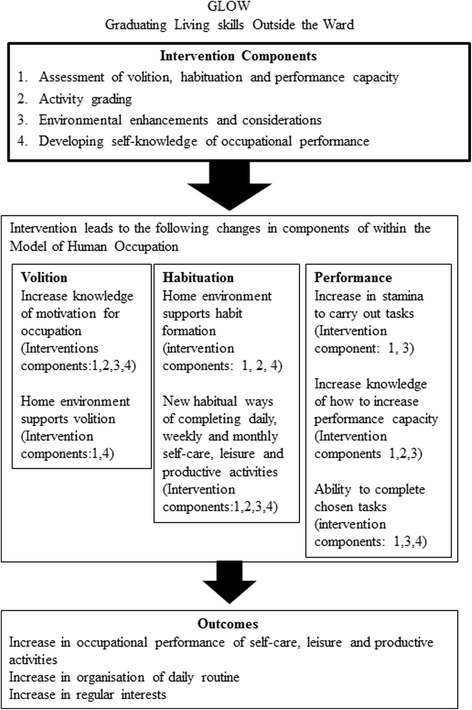
Process of change in GLOW

As can be seen in Fig. 3, the outcomes of GLOW have been identified as an increase in occupational performance of self-care and leisure, organisation of daily routine and an increase in regular leisure interests. These have been measured in two feasibility studies using measures of time-use and satisfaction in time use and social functioning. Distal outcomes of reduction in admissions and crisis service use and an increase in quality of life and wellbeing and have also been identified. These outcomes have also been measured in one of feasibility studies by counting the number of admissions and the time to readmission before, during and at a follow-up time point after the intervention had finished. Measures of quality of life and wellbeing have also been used to measure change during the feasibility studies. These studies will be published separately.

In summary, through the use of the Medical Research Council (MRC) framework to guide the study, new primary research was carried out involving service users and clinicians to develop a manualised occupational therapy intervention called GLOW for use following discharge from hospital.

## Discussion

This intervention development study describes the rationale, methods and process of the development of an intervention, GLOW, using the MRC framework for developing and evaluating complex interventions as guidance. The development of this intervention serves as an example of how to develop an intervention where the problem of the intervention should focus on is not already identified and described in the literature, and new knowledge generation is required. Use of the MRC framework in this study ensures the intervention developed is empirically defensible and developed sufficiently to be tested in a feasibility study. The MRC framework offered a formal and recognised structure to the development of the intervention, and this rigorous approach resulted in the development of an evidence-based intervention with a theoretical underpinning. Use of this structured approach and manualisation of the intervention supports internal validity of a future randomised controlled trial at the evaluation stage of the intervention within the MRC framework [39].

### Strengths and limitations

Since the development of GLOW, there has been an increase in knowledge and formal frameworks regarding health intervention development, for example, Intervention Mapping [40, 41]. These frameworks provide more detail regarding the process of identifying the intervention components and how they target the health problem the intervention aims to improve, and can be used in the development of future occupational therapy interventions.

#### What this study has added

This study reported the development of an intervention for people with psychotic disorder. The developed intervention, GLOW, has the potential to improve the efficiency of delivery of community mental health services as it is time limited and manualised it can increase number of patients seen by occupational therapists. The manualised nature and 1-day training of the intervention support its potential for wider application across the health service. Two feasibility studies of GLOW with people with psychosis, and with people with a mood or personality disorder, which will be published separately, have demonstrated the acceptability and feasibility of its use in clinical practice as well as suitability of the intended outcomes for full evaluation. This has been identified as a crucial step in optimising the intervention prior testing the effectiveness of the intervention [42].

#### Next steps

The next step is the preparation of a trial protocol to evaluate the clinical and cost effectiveness of GLOW in a randomised controlled trial at the evaluation phase of the MRC framework.

## Conclusions

This intervention development study has described the development of an intervention for people with a diagnosed psychotic disorder to improve occupational performance following discharge from hospital.

Through the use of the MRC framework to guide the study, new primary research was carried out involving service users and clinicians to develop a manualised evidence-based occupational therapy intervention called GLOW for use following discharge from hospital.

The next phase for the intervention is to test the clinical and cost effectiveness in a randomised controlled trial.

## Additional file

Additional file 1: A summary of the studies included in the review. (DOCX 16 kb)

## Abbreviations

GLOW: Graduating Living skills Outside the Ward
IPS: Individual Placement and Support
IRM: Intentional Relationship Model
MOHO: Model of Human Occupation
MOHOST: Model of Human Occupation Screening Tool
MRC: Medical Research Council
REIS-SF: Residential Environment Impact Scale-Short Form
TIDieR: Template for Intervention Description and Replication
