# Supplementary material for: The development of an occupational therapy intervention for adults with a diagnosed psychotic disorder following discharge from hospital
Source: Pilot Feasibility Stud. 2018 Apr 23;4:81. doi: 10.1186/s40814-018-0267-7 (PMC5914033; doi:10.1186/s40814-018-0267-7)
Supplement: Supplementary file 1 — A summary of the studies included in the review. (DOCX 16 kb) [file 40814_2018_267_MOESM1_ESM.docx]

| Author, Year | Intervention | Intervention reproducible | Design | Population | Primary outcome | Quality Rating |
| --- | --- | --- | --- | --- | --- | --- |
| Buchain et al, 2003 [1] | Occupational therapy and clozapine | No intervention manual  No theoretical base | RCT | Adults with treatment resistant schizophrenia | Scale for Interactive Observation in occupational therapy (EOITO) | Weak |
| Brown et al, 2002 [2] | Improving grocery shopping skills | No intervention manual  No theoretical base | Pre-test  Post-test | Adults with schizophrenia or schizoaffective | Test of Grocery Shopping Skills | Weak |
| Cook et al 2009 [3] | Occupational Therapy for people with psychosis in community setting | No theoretical base | RCT | Adults with diagnosed psychotic disorder of any duration | Social Functioning Scale (SFS) | Strong |
| Cook & Howe 2003 [4] | Occupational Therapy: based on PEOP model | No intervention manual | Pre-test  post-test | Adults with psychosis | Social Functioning Scale (SFS) | Strong |
| Duncombe 2004 [5] | Learning of a functional living skill- cooking, clinic compared to home | No intervention manual  No theoretical base | Non- randomised Controlled trial | Adults with schizophrenia or schizoaffective disorder for at least 5 years | Allen Cognitive Level Screen  ( ACLS-90)  Kitchen Task Assessment-Modified | Weak |
| Grimm et al, 2009 [6] | Acquisitional frame of reference (FOR) and psycho educational to improve meal preparation | No intervention manual | RCT | Adults with diagnosis of subtype of schizophrenia or schizoaffective | Performance Assessment of Self-Care Skills (PASS) | Weak |
| Liberman et al 1998 [7] | Life skills training | No theoretical base | RCT | Adults with persistent forms of schizophrenia | Independent Living Skills Survey (ILSS) | Moderate |
| Mairs & Bradshaw 2004 [8] | Life skills programme | Model of Functional deficits developed by author | Pre-test  Post-test | Adults with diagnosis of schizophrenia or schizoaffective | Social Functioning Scale (SFS) | Strong |
| Porter et al, 2000 [9] | An individualised food-skills programme | No intervention manual  No theoretical base | An individualised food-skills programme | Adults with schizophrenia for over 20years | Functional Needs Assessment- Nutritional Management Programme  (FNA-NMP) | Moderate |
| Raweh & Katz, 1999 [10] | Intervention based on Allen’s Cognitive Disabilities Model | No intervention manual | Non-randomised controlled trial | Adults in post acute stage of schizophrenia | Routine Task Inventory (RTI-2) | Weak |
| Schindler, 2008  [11] | Role development to increase task and interpersonal skills and social roles | Untested theoretical base | Pre-test  Post-test  Case study | Adults with diagnosis of schizophrenia from 4yrs to 20yrs duration | Role Functioning Scale | Moderate |

1. Buchain PC, Vizzotto ADB, Henna Neto J, Elkis H. Randomized controlled trial of occupational therapy in patients with treatment-resistant schizophrenia. Rev. Bras. Psiquiatr. 2003;*25*:26-30.
2. Brown C, Rempfer M, Hamera E. Teaching Grocery Shopping Skills to People with Schizophrenia. Occup Ther J Res. 2002;22:90S-91S.
3. Cook, S. What helps and hinders people with psychotic conditions doing what they want in their daily lives. Br J Occup Ther. 2009;2:248.
4. Cook S, Howe A. Engaging people with enduring psychotic conditions in primary mental health care and occupational therapy. Br J Occup Ther. 2003;66:236-46.
5. Duncombe LW. Comparing Learning of cooking in Home and clinic for People with Schizophrenia. Am J Occup Ther. 2004;58:272-278.
6. Grimm E Z, Meus JS, Brown C, Exley SM, Hartman S, Hays C & Manner T. Occup Ther J Res. 2000;29:148-153.
7. Liberman R P, Wallace C J, Blackwell G, Kopelowicz A, Vaccaro J V, Mintz J. Skills training versus psychosocial occupational therapy for persons with persistent schizophrenia. Am J Psychiatry. 1998;155(8):1087-91.
8. Mairs H, Bradshaw T. Life skills training in schizophrenia. Br J Occup Ther. 2004; 67:217-24.
9. Porter J, Capra S, Watson G. An individualized food-skills programme: Development, implementation and evaluation. Aust Occup Ther J. 2000;47:51-61.
10. Raweh DV, Katz N. Treatment effectiveness of Allen's cognitive disabilities model with adult schizophrenic outpatients: a pilot study. Occup Ther Ment Health .1999;14:65-77.
11. Schindler VP. Developing Roles and Skills in Community Living Adults with Severe and Persistent Mental Illness. Occup Ther Ment Health. 2008;24:135-153.
